# Macrophage Lysophosphatidylcholine Acyltransferase 3 Deficiency-Mediated Inflammation Is Not Sufficient to Induce Atherosclerosis in a Mouse Model

**DOI:** 10.3389/fcvm.2018.00192

**Published:** 2019-01-17

**Authors:** Hui Jiang, Zhiqiang Li, Chongmin Huan, Xian-Cheng Jiang

**Affiliations:** ^1^Department of Cell Biology, State University of New York Downstate Medical Center, Brooklyn, NY, United States; ^2^Molecular and Cellular Cardiology Program, VA New York Harbor Healthcare System, Brooklyn, NY, United States; ^3^Department of Surgery, State University of New York Downstate Medical Center, Brooklyn, NY, United States

**Keywords:** lysophosphatidylcholine acyltransferase 3 (LPCAT3), phosphatidylcholine remodeling, macrophage Lpcat3 gene knockout mice, inflammation, atherosclerosis

## Abstract

Mammalian cell membrane phosphatidylcholines (PCs), the major phospholipids, exhibit diversity which is controlled by Lands' cycle or PC remodeling pathway. Lysophosphatidylcholine acyltransferase (LPCAT) is one of the major players in the pathway and plays an important role in maintaining cell membrane structure and function. LPCAT3 is highly expressed in macrophages, however, its role in mediating inflammation is still not understood, since contradictory results were reported previously. The order of LPCAT mRNA levels in mouse macrophages is as follows: LPCAT3 > LPCAT1 > LPCAT2 >> LPCAT4. In order to investigate the role of *LPCAT3* in macrophages, we prepared myeloid cell-specific *Lpcat3* knockout (KO) mice and found that the deficiency significantly reduced certain polyunsaturated phosphatidylcholines, such as 16:0/20:4, 18:1/18:2, 18:0/20:4, and 18:1/20:4 in macrophage plasma membrane. *Lpcat3* deficiency significantly increased toll like receptor 4 protein and phosphorylated c-Src in membrane lipid rafts, and increased LPS-induced IL-6 and TNFα releasing through activation of MAP kinases and NFκB. Moreover, the ablation of LPCAT3 in macrophages significantly increase of M1 macrophages. However, macrophage deletion of *Lpcat3* in (LDL receptor) *Ldlr* KO mice, both male and female, on a Western type diet, did not have a significant impact on atherogenesis. In conclusion, LPCAT3 is one of LPCATs in macrophages, involved in PC remodeling. LPCAT3 deficiency has no effect on cholesterol efflux. However, the deficiency promotes macrophage inflammatory response, but such an effect has a marginal influence on the development of atherosclerosis.

## Introduction

Phosphatidylcholines (PCs), the major phospholipids, on mammalian cell membrane exhibit structural diversity (1, 2). Polyunsaturated PCs ensure the fluidity of cell membrane. In macrophages, the plasma membrane provides a platform that mediates inflammation. lipopolysaccharide (LPS) or peptidoglycan treatment promotes the assembly of the toll like receptor (TLR) complex in lipid rafts (3–5). We found that a decrease in macrophage plasma membrane sphingomyelin level can effectively prevent inflammatory responses by reducing TLR4 expression (6–8), thus decreasing atherosclerosis (6, 7, 9). It is also reported that cellular lipids are important regulators of c-Scr activation by altering the recruitment of C-Scr to the plasma membrane (10) and many studies also have shown a critical role for c-Src in macrophage-mediated inflammatory responses (11). It is known that the composition of polyunsaturated PCs in membranes is regulated by LPCATs (12–14).

There are four isoforms for LPCAT (13, 15–18). The major isoform in the liver and macrophage is LPCAT3 (14, 18–20). LPCAT3 exhibits an acyl donor preference toward polyunsaturated fatty acid-CoA molecules like arachidonoyl-CoA (18, 21). Modifications of polyunsaturated PC composition on cell membrane have an impact on many biological processes (22–27). We found that *Lpcat3* deficiency significantly reduces polyunsaturated PCs on the hepatocytes and enterocytes and impacts plasma lipid metabolism (28).

The development of atherosclerosis is closely related with inflammation. Macrophage-derived foam cells in the vessel wall can produce many pro-inflammatory chemokines and cytokines (29) which promote atherogenesis. Previously, one study showed that *Lpcat3* silencing significantly increased LPS-mediated inflammatory response in macrophages and this could be due to the decrease of macrophage membrane polyunsaturated PCs (14). On the contrary, another study indicated that *Lpcat3* silencing did not influence macrophage LPS-induced inflammatory response, although PC composition changes were also observed (19). We still do not understand the discrepancy of both studies and still do not know whether PC remodeling in macrophage has an impact on inflammation. Very recently, it has been reported that *Lpcat3* deficiency in hematopoietic cells influence cholesterol and phospholipid metabolism and promotes atherosclerosis in a mouse model (30). However, macrophage specific *Lpcat3* deficiency on atherosclerosis is still not precisely evaluated. In this study, we utilized myeloid cell-specific *Lpcat3* deficient mice to study the effect of *Lpcat3* deficiency on cholesterol efflux, inflammation, and atherosclerosis. We hypothesized that alterations in the levels of macrophage membrane polyunsaturated PCs affect membrane fluidity, cholesterol efflux and inflammatory responses.

## Materials and Methods

### Generation of Myeloid Cell-Specific *Lpcat3*-Deficient Mice

**Figure 1:**
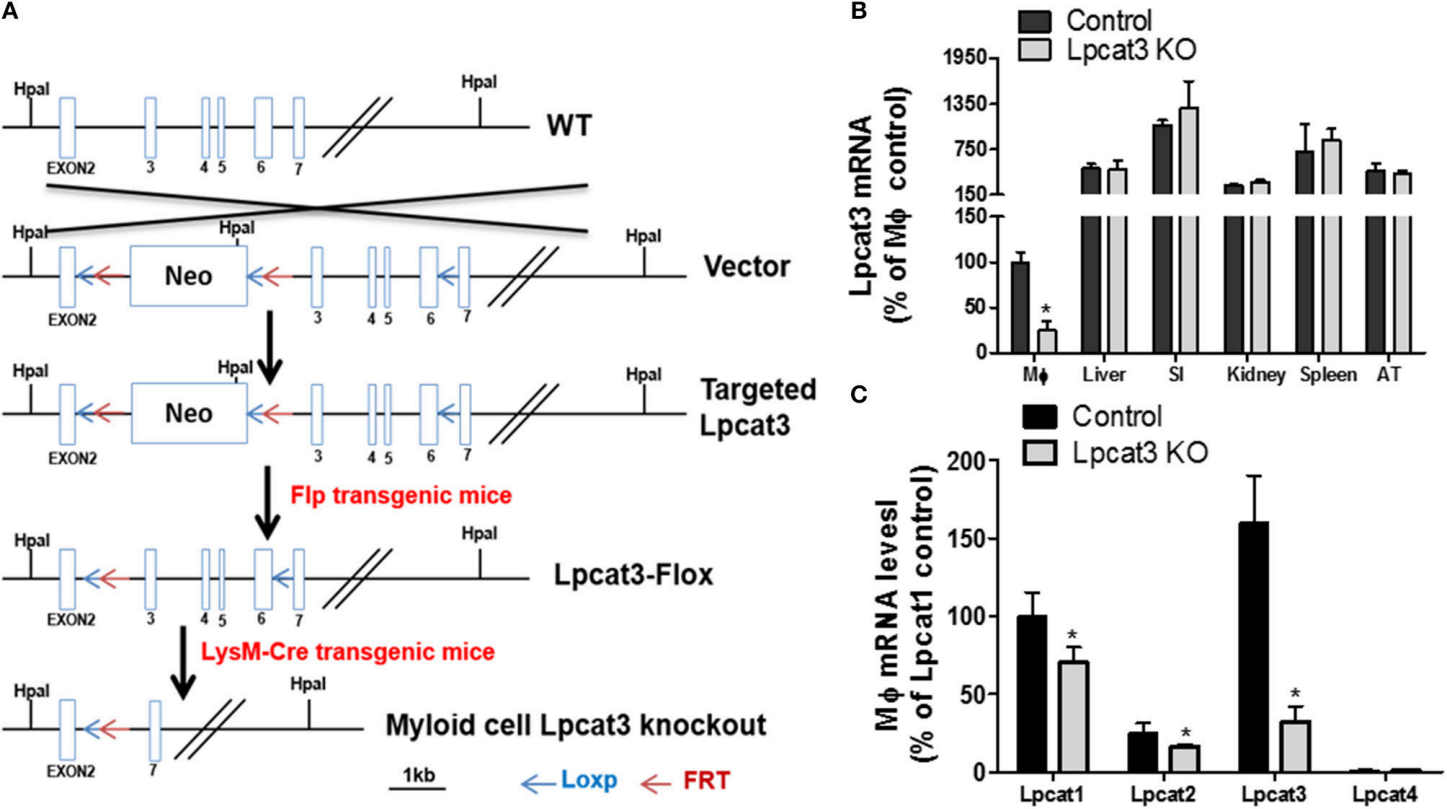
Myeloid cell-specific *Lpcat3* KO mouse preparation. **(A)** Strategy for myeloid cell-specific *Lpcat3*-deficient mice preparation. **(B)** LPCAT3 mRNA tissue distribution. (SI, small intestine; AT, adipose tissue) **(C)** Macrophage LPCAT1, LPCAT2, and LPCAT3 mRNA tissue distribution. mRNAs were measured by Real-time PCR. Values are mean ± SD, *n* = 4, ^*^P < 0.01.

*Lpcat3*-Flox mice (28) were crossed with LysM-Cre transgenic mice (Jackson Laboratory) to establish *Lpcat3*-Flox/LysM-Cre mice according to the strategy (Figure 1A). We used both male and female mice, with C57BL/6 background and at age of 12-week-old. Our studies were approved by the Institutional Animal Care and Use Committee of State University of New York Downstate Medical Center.

### Bone Marrow-Derived Macrophage Isolation

Mice were sacrificed by CO_2_. Bone marrow cells were isolated and macrophages were cultured as we did before (7). To eliminated the effect of FBS on macrophage surface PC composition, medium was changed to serum-free medium (0.2% BSA DMEM) for 24 h before all *in vitro* experiments.

### mRNA Measurement

Total RNA was extracted from the cells using Trizol method (Invitrogen). The Superscript™ III First-strand Synthesis kit (Invitrogen) was used for cDNA synthesis. SYBR Select Master Mix kit (Applied Biosystems) was used for PCR with following program: activation at 95°C for 10 min followed by 40 amplification cycles of 95°C for 15 s and 60°C for 60 s. The gene encoding Gapdh was used as internal controls. Relative gene expression is expressed as the mean ± SD. Mouse *Lpcat3* primers: forward, TTTCTGGTTCCGCTGCATGT, reverse, CCGACAGAATGCACACTCCTTC; *Gapdh* primers: forward, TGTAGACCATGTAGTTGAGGTCA; reverse, AGGTCGGTGTGAACGGATTTG. *Lpcat1* primers: forward, CGTGAATATGTGGTCGCCTTG, reverse, ATGCTGCCATCCTCAGGAGAT. Lpcat2 primers: forward, GTCCAGCAGACTACGATCAGTG, reverse, CTTATTGGATGGGTCAGCTTTTC. Lpcat4 primers: forward, TTCGGTTTCAGAGGATACGACAA, reverse, AATGTCTGGATTGTCGGACTGAA.

### Measurement of Total LPCAT3 Activity and PC Subspecies

LPCAT3 activity was measured according to a published protocol, using NBD-lysoPC and arachidonoyl-CoA as subtracts (20). Liquid chromatography-coupled tandem mass spectrometry (LC-MS/MS) was used for the measurement of PC subspecies as described (20).

### M1/M2 Measurement

Control and *Lpcat3* KO mice were euthanized by CO_2_, and peritoneal macrophages were harvested by washing abdominal cavity with cold PBS. Harvested peritoneal resident macrophages were made to single-cell suspensions. Cells were then blocked with 0.5% BSA (w/v) and 2% FBS (v/v) in PBS and then stained with antibodies F4/80 (1:600 dilutions; BD Bioscience), CD11b (1:600 dilutions; BD Bioscience), and CD80 (1:600 dilutions; BD Bioscience) or CD206 (1: 400; Thermo Fisher). After being washed 3 times, cells were suspended in PBS and analyzed by Flow-cytometery.

### Western Blot for Macrophage Lipid Rafts Lyn, TLR4, Total c-Scr, and Phosphorylated c-Scr

Macrophages (50 × 10^6^), derived from Bone marrow, were homogenized. A previously reported method was used for lipid rafts isolation (8). Equal amount of protein from all fragments were used for Western blots with specific antibodies to Lyn (Santa Cruz), TLR4 (Santa Cruz), total c-Scr (Cell Signaling), and phospho-Src-Tyr416 (Cell Signaling).

### TNF-α and IL-6 Measurements

Bone marrow–derived macrophages were treated with 10 ng/ml LPS for 16 h and TNF- α and IL-6 released to the medium were analyzed with ELISA kits (eBiosciences).

### Western Blot for Macrophage p38 and p42/44

To eliminate lipoprotein effect from FBS to cell surface PC composition, Bone marrow-derived macrophage from Control and *Lpcat3* KO mice were changed to serum-free medium 24 h before experiment. Macrophages were then treated with 1 μg/ml LPS in 0.2% BSA DMEM for 0, 10, and 20 min. Cells were washed with cold PBS and harvested. Cells were homogenized in TSE buffer (50 mM Tris, 200 mM NaCl, 1 mM EDTA, pH 7.5). Cell lysates were used for Western blots with antibodies against p38 and p42/44 (Cell Signaling). The maximum intensity of each band was measured by Image-Pro Plus version 4.5 software (Media Cybernetics Inc.).

### Nuclear Preparation and Western Blot for P65

We isolated macrophage nucleus using a Kit (Thermo Scientific). The nuclear preperation was utilized for Western blot with specific antibodies to p65 (Santa Cruz) and anti-histone 3 (H3).

### Isolation of Lipid Rafts

Bone marrow-derived macrophages (50 × 10^6^) were homogenized and lipid rafts were isolated by a previously reported method (8).

### Cholesterol Efflux (*ex vivo*) Measurement

Bone marrow-derived macrophages were labeled with [^3^H]cholesterol carried by acetylated-LDL. The cholesterol efflux was measured by an established method (7).

### Bone Marrow Transplantation and Atherosclerosis Study Model

*Ldlr* KO female or male mice (age 8 weeks, Jackson Laboratory) were utilized. Bone marrow transplantation was performed as previously described (7). After 8 weeks transplantation, all mice were on a high fat high cholesterol diet for 3 months.

### Mouse Atherosclerotic Lesion Measurement

We used the method which we reported before for atherosclerotic lesion measurement (7).

### Statistical Analysis

Mean ± SD is expressed for each results. Data between two groups were analyzed by the unpaired, two-tailed Student's *t*-test, and among multiple groups by ANOVA followed by the Student-Newman-Keuls (SNK) test.

## Results

### Production of Myeloid Cell-Specific *Lpcat3*-Deficient Mice

**Figure 2:**
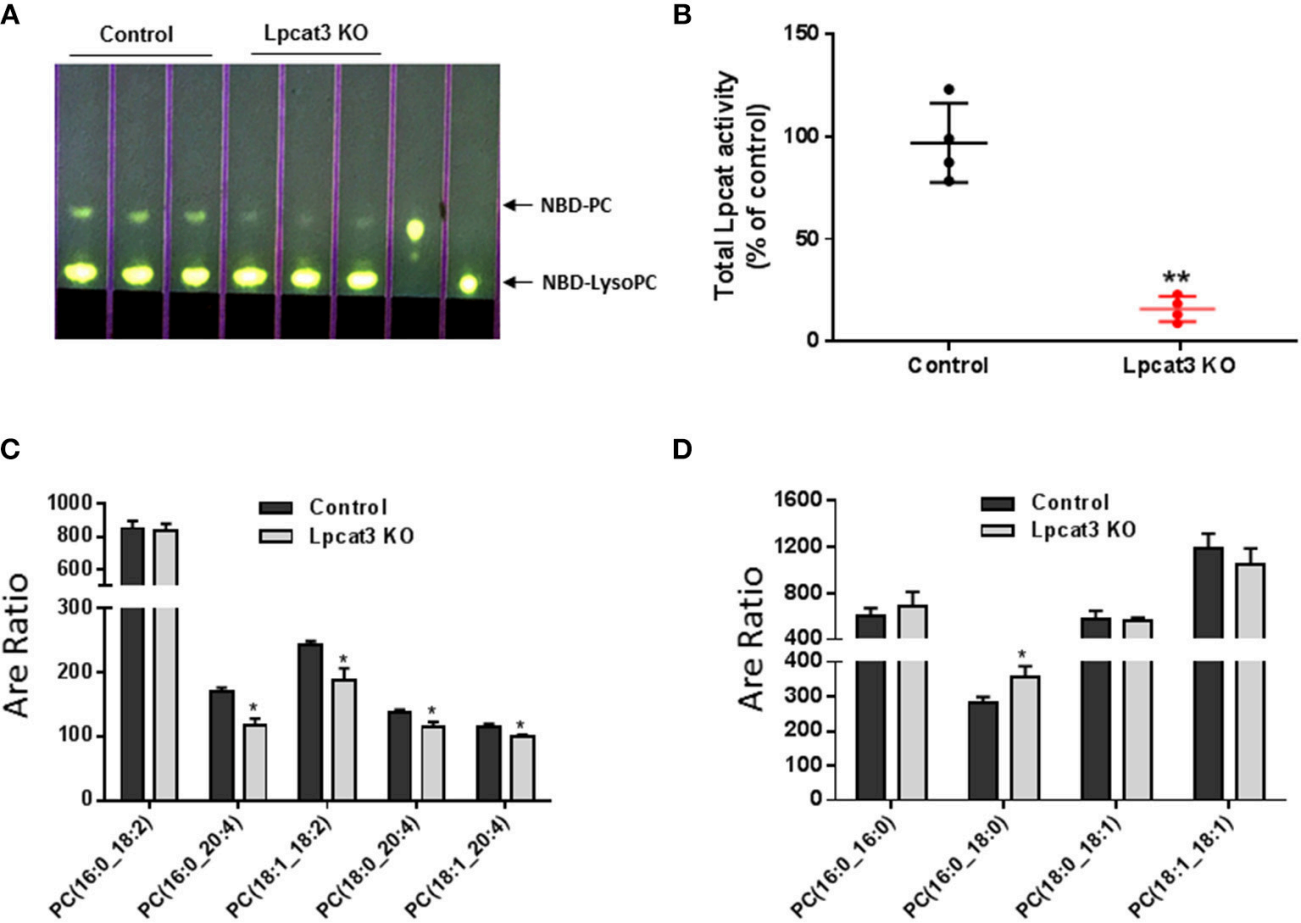
LPCAT3 activity and PC composition measurements. **(A,B)** LPCAT3 activity measurement and quantification. The detail procedure was stated in “Materials and Methods.” **(C,D)** PC subspecies measured by LC/MS/MS. Values are mean ± SD, *n* = 3–4, ^*^P < 0.05, ^**^P < 0.01.

To produce myeloid cell-specific *Lpcat3* KO mice, we crossed *Lpcat3*-Flox mice with LysM-Cre transgenic mice. We collected bone marrow derived macrophage, liver, small intestine, adipose tissue, kidney, spleen from the homozygous KO male mice. Compared with controls, *Lpcat3* mRNA level was decreased by 80% in the macrophage but no other tissues (Figure 1B). We also noticed that among the tested tissues, macrophage is the lowest one for LPCAT3 expression (Figure 1B). Moreover, we found that, besides LPCAT3, LPCAT1, and LPCAT2 are also LPCAT isoforms expressed in macrophages and both may also play important roles in PC remodeling in the cell. This is different from the liver and small intestine, where LPCAT1, LPCAT2, and LPCAT4 are negligible (28). Compared with controls, the mRNA levels of LPCAT1 and LPCAT2 were significantly reduced by 30 and 35%, respectively, in *Lpcat3* KO macrophages (Figure 1C). We then measured total LPCAT3 activity using NBD-lysoPC and arachidonoyl-CoA (14) in the macrophage homogenate and found it was decreased by 80% compared with controls (Figures 2A,B). Similar results were obtained with female mice (data not shown).

### Effect of *Lpcat3* Deficiency on Macrophage Inflammation, Cholesterol Efflux, and ER Stress

PC subspecies populations in macrophage homogenates were analyzed by LC/MS/MS. We found that *Lpcat3* deficiency decreased the amount of polyunsaturated PCs (16:0/20:4, 18:1/18:2, 18:0/20:4, and 18:1/20:4) in the membrane (Figure 2C), while other PCs have no significant changes except 16:0/18:0 which was increased (Figure 2D). These changes could affect macrophage mediated inflammatory response.

**Figure 3:**
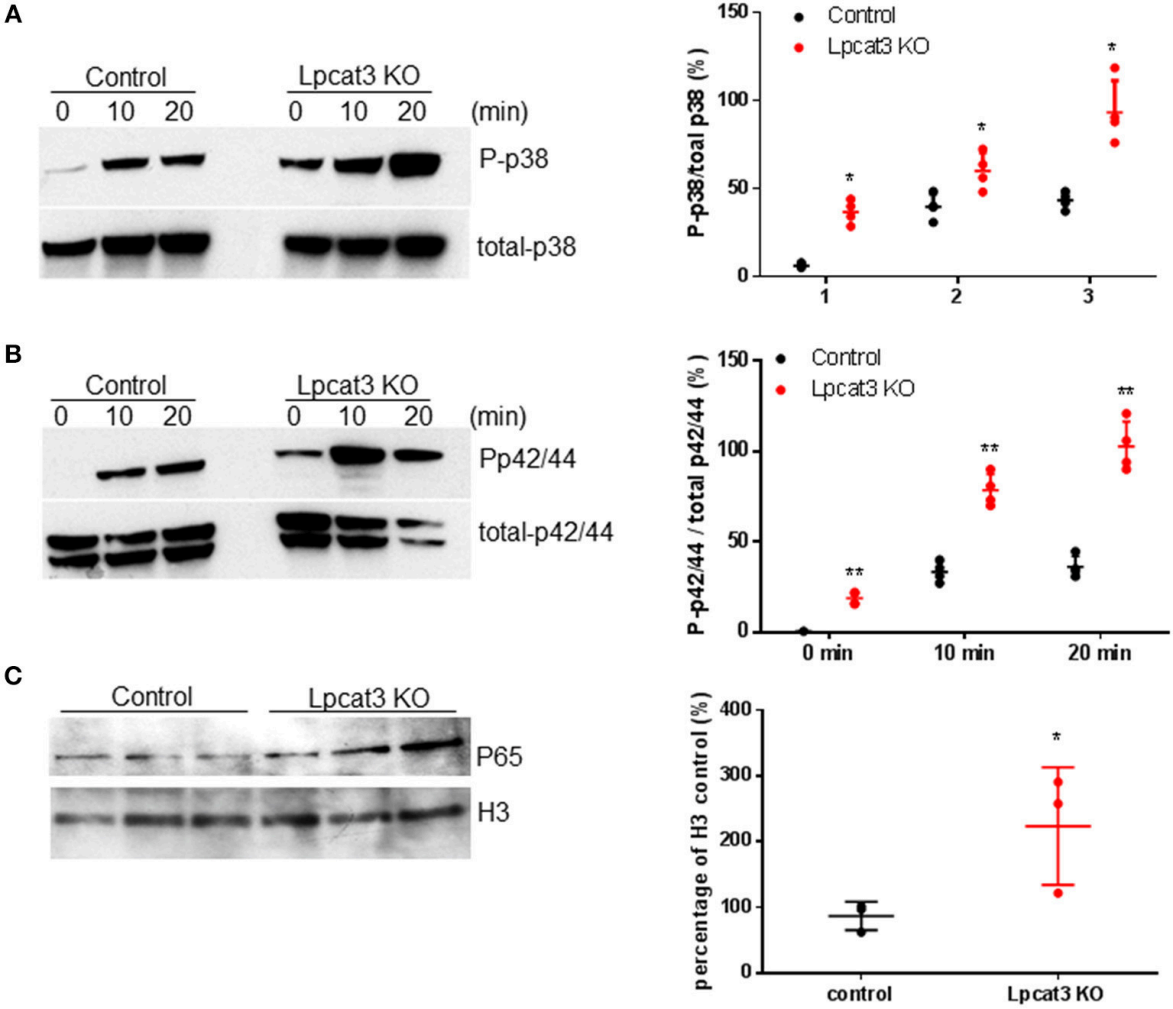
*Lpcat3* KO macrophages promotes p38, p42/44, and NFκB activation. WT and *Lpcat3* KO macrophages were treated with 1 μg/ml LPS. **(A)** Western blot and quantitative display of total p38 and phosphorylated p38. **(B)** Western blot and quantitative display of total p42/44 and phosphorylated p42/44. Macrophage nucleus were isolated and p65 was measured by Western blot. **(C)** Western blot and quantitative display of p65. Values are mean ± SD, *n* = 3–4, ^*^P < 0.01; ^**^P < 0.01.

We investigated to consequence of *Lpcat3* deficiency in macrophage inflammatory responses. After LPS (1 μg/ml) treatment, *Lpcat3* KO macrophages significantly increased levels of phosphorylated p38 and p42/44 (Figures 3A,B). We also measured nucleus NFκB subunit p65 and found it was increased in *Lpcat3* KO macrophages (Figure 3C).

**Figure 4:**
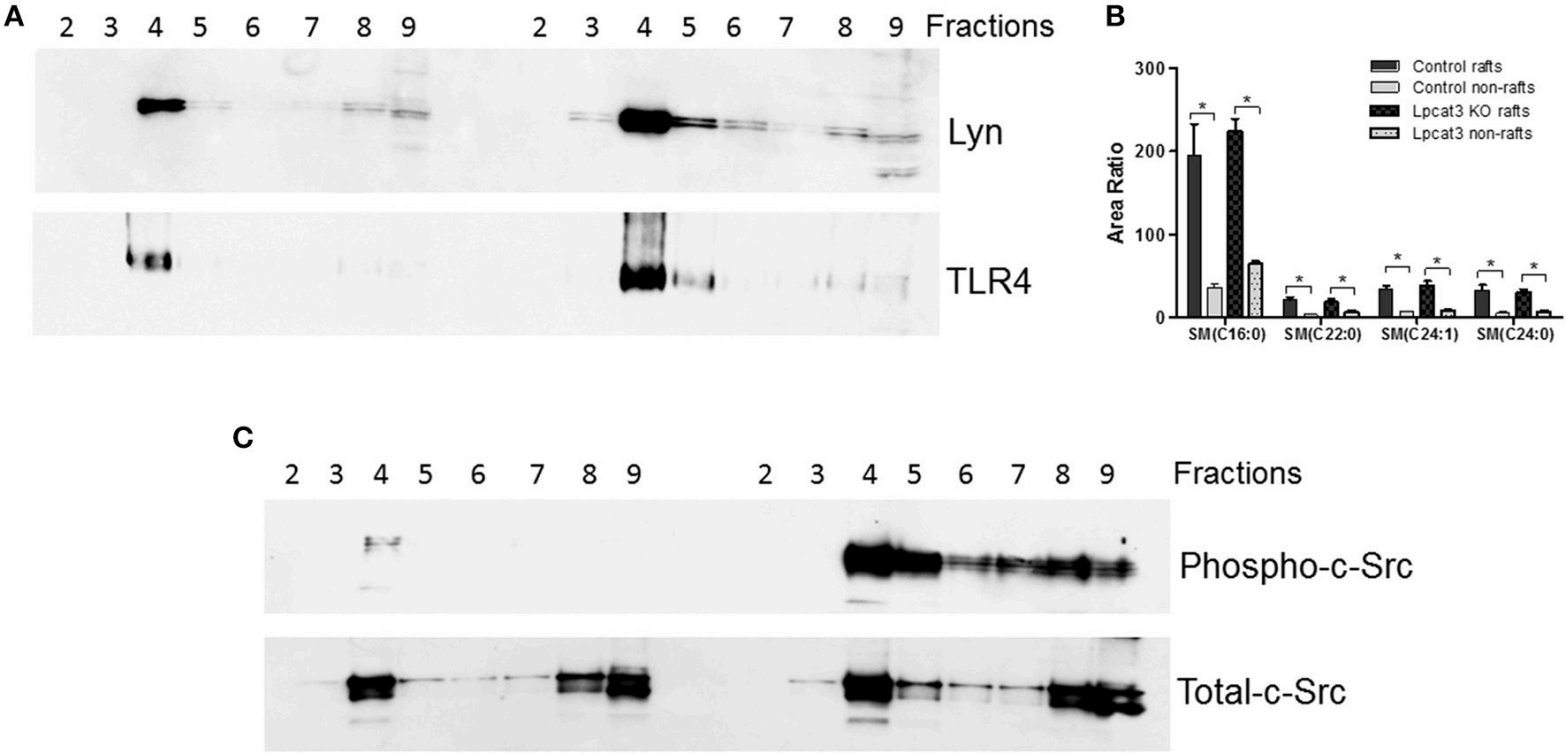
Lipid rafts TLR4 and phosphorylated c-Src measurement. Macrophage lipid rafts isolated according a method in “Materials and Methods.” **(A)** Western blot of Lyn Kinase (a lipid rafts marker) and TLR4 in each fractions from lipid rafts isolation. **(B)** LC/MS/MS measurement for sphingomyelin levels in rafts and non-rafts regions. **(C)** Western blot of phosphorylated c-Src and total c-Src in each fractions from lipid rafts isolation. The picture is the representative of three experiments. ^*^p < 0.05.

We then sought to investigate TLR4 levels in the KO macrophages and controls, since TLR4 is upstream of NFκB and MAP kinase. Lipid rafts play essential role in TLR4-mediated signaling (4, 31), thus, we examined whether *Lpcat3* deficiency affects TLR4 levels in the lipid rafts. We isolated lipid rafts which are enriched with Lyn kinase (marker of lipid rafts) (Figure 4A) and different subspecies of sphingomyelin (Figure 4B), as reported before (7). As seen on Figure 4A, lipid raft regions contain much more TLR4 protein compared with controls.

A recent report indicated that c-Src phosphorylation (activation)-mediated NFκB activation and then TNFα elevation could participate in macrophage activation and inflammation (32). We found that *Lpcat3* deficiency dramatically increased phosphorylated c-Src in macrophage lipid rafts (Figure 4C) as assessed by Western blot using phospho-Src-Tyr416 antibody.

**Figure 5:**
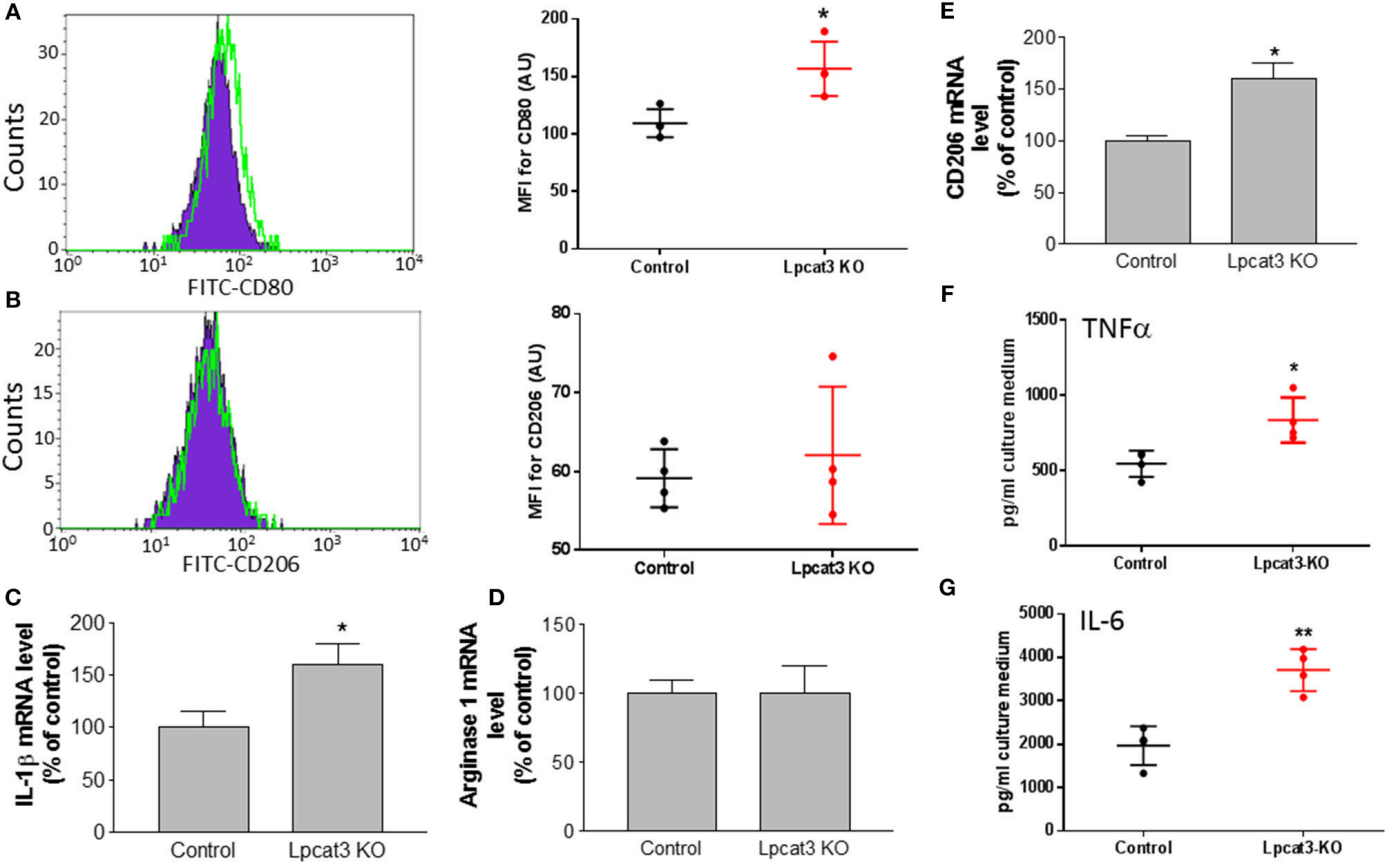
Evaluation of inflammation in *Lpcat3* KO macrophages. **(A,B)** M1/M2 macrophage measurement. Harvested peritoneal macrophages were made to single-cell suspensions. Cells were stained with antibodies F4/80, CD11b, and CD80 or CD206. Cell suspension was analyzed by Flow cytometry. **(C)** Macrophages were treated 10 ng/ml LPS for 16 h, IL-1β mRNA was measured. **(D,E)** Macrophages were treated 20 ng/ml IL-4 for 24 h, arginase-1, and CD206 mRNAs were measured, respectively. **(F,G)** Macrophages were treated with 10 ng/ml LPS for 16 h and TNF-α and IL-6 released to the medium were analyzed with ELISA kits (eBiosciences). Values are mean ± SD, *n* = 3–4, ^*^P < 0.01; ^**^P < 0.01.

To further confirm the impact of macrophage *Lpcat3* deficiency mediated inflammation, we utilized F4/80, CD11b, and CD80 antibodies to label M1 and F4/80, CD11b, and CD206 antibody to label M2 macrophages, respectively, then measured abundancy of both macrophages using Flow cytometry. We found that *Lpcat3* deficiency significantly increased M1 (Figure 5A) but not M2 macrophages (Figure 5B). We further treated macrophages with LPS and IL-4, respectively. We found that LPS treatment significantly increased IL-1β mRNA levels in the deficient macrophages (Figure 5C), while IL-4 treatment had no effect on arginase1 (Figure 5D) but increased CD206 mRNA levels (Figure 5E).

We also determined the impact of the deficiency-related proinflammatory cytokine production. We stimulated the macrophage with LPS and found that *Lpcat3* deficiency significantly promotes IL-6 and TNFα secretion from macrophages, compared with controls (Figures 5F,G).

**Figure 6:**
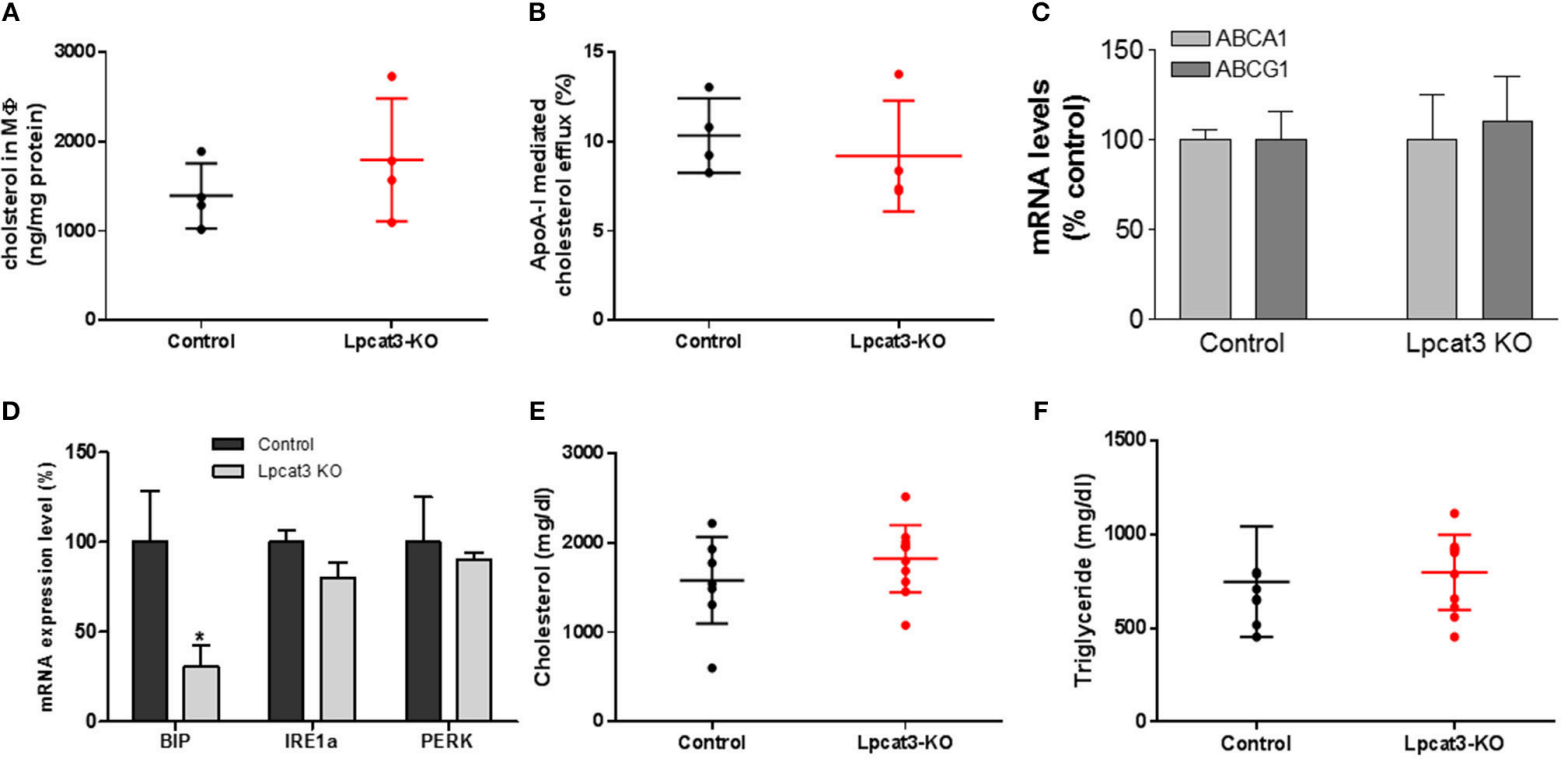
Measurements of cholesterol loading and efflux, ER stress markers, and plasma lipids. WT and *Lpcat3* KO bone marrow derived macrophages were labeled with [^3^H]cholesterol carried by acetylated-LDL. **(A)** Cellular cholesterol measurement after acetylated-LDL loading. **(B)** ApoA-I mediated cholesterol efflux. **(C)** mRNA levels of ABCA1 and ABCG1. **(D)** mRNA levels of ER stress markers (BIP, IRE1α, and PERK) were measured by real-time PCR. **(E,F)** Plasma cholesterol and triglyceride levels in WT → *Ldlr* KO and *Lpcat3* KO → *Ldlr* KO mice. Values are mean ± SD, *n* = 4–8, ^*^P < 0.01.

A previous study indicated that fetal liver derived *Lpcat3* deficient macrophage reduced cholesterol efflux (30). We first utilized Ac-LDL to load bone marrow derived macrophages with cholesterol and we did not find a difference in cholesterol accumulation between control and *Lpcat3* deficiency (Figure 6A). We then utilized Ac-LDL and [^3^H]-cholesterol to load the cells with [^3^H]-cholesterol and then evaluated cholesterol efflux using apoA-I. We also did not find significant difference between control and *Lpcat*3 deficiency (Figure 6B). We further measured mRNA levels of ABCA1 and ABCG1, both transporters are involved in cholesterol efflux, and we did not find any significant changes (Figure 6C).

It has been reported that *Lpcat3* knockdown in macrophages exacerbated mRNAs of genes which are involved in ER stress (14). We measured mRNA levels of BIP, IRE1α, and PERK in control and *Lpcat3* KO microphages and found that BIP mRNA was significantly reduced while mRNA levels of IRE1α and PERK had no significant difference (Figure 6D).

### Bone Marrow Transplantation and Atherosclerosis Evaluation

To evaluate the impact of the *Lpcat3* deficiency on atherosclerosis, we transplanted *Lpcat3* KO or wide type (WT) bone marrow into lethally irradiated *Ldlr* KO mice to produce *Lpcat3* KO → *Ldlr* KO (experimental) and WT → *Ldlr* KO (control) mice. We then fed the animal with a high fat high cholesterol diet (0.15% cholesterol, 20% saturated fat) for 3 months. We found no significant changes in plasma lipid cholesterol and triglyceride levels (Figures 6E,F). We also found that there was no difference in body weight gain in these animals (data not shown).

**Figure 7:**
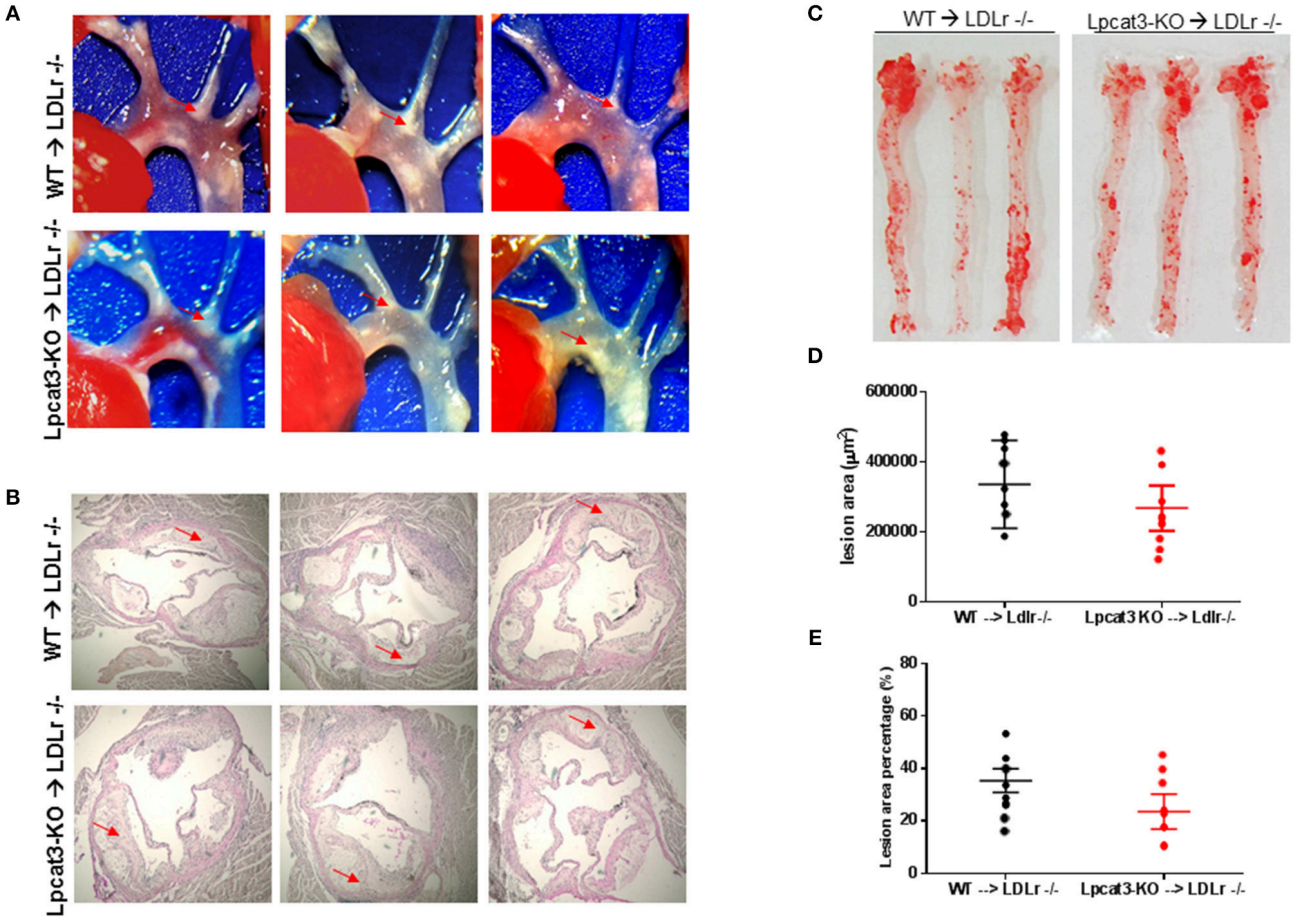
Atherosclerosis measurement in male WT → *Ldlr* KO and *Lpcat3* KO → *Ldlr* KO mice. **(A)** Aortic arches with atherosclerotic plaques (white areas). **(B)** Aortic root assay for lesion areas after H&E staining. Six alternate sections (10 μm thick) sliced from paraffin-fixed aortic root tissues of each transplanted mouse were used for the analysis. **(C)** *En face* aortic plaque analysis after Oil Red O staining. **(D)** Quantitative display of root assay. **(E)** Quantitative display of *en face* assay. Red arrows indicate lesion area. Values are mean ± SD. *N* = 8–9.

**Figure 8:**
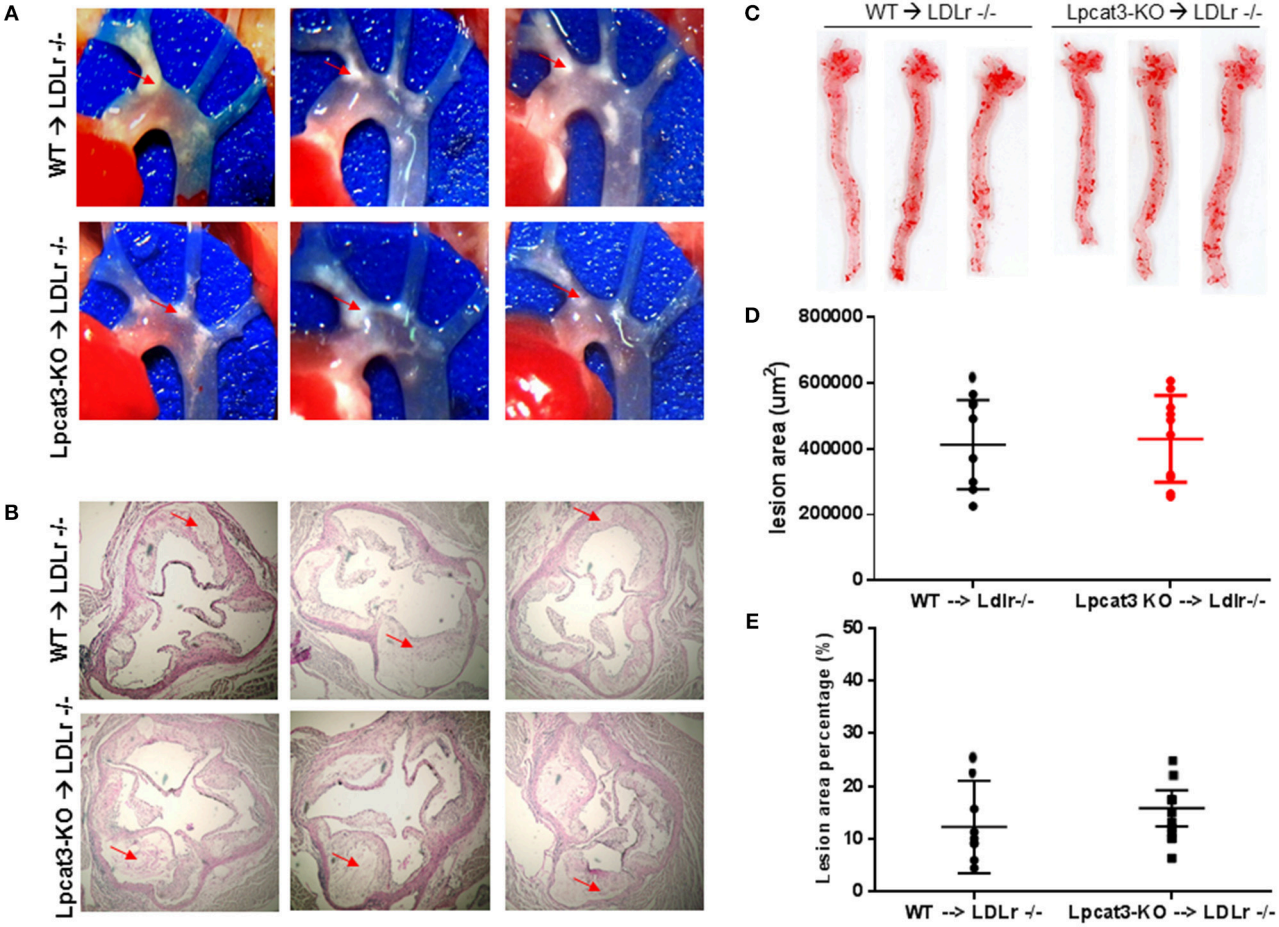
Atherosclerosis measurement in female WT → *Ldlr* KO and *Lpcat3* KO → *Ldlr* KO mice. **(A)** Aortic arches with atherosclerotic plaques (white areas). **(B)** Aortic root assay for lesion areas after H&E staining. Six alternate sections (10 μm thick) sliced from paraffin-fixed aortic root tissues of each transplanted mouse were used for the analysis. **(C)** *En face* aortic plaque analysis after Oil Red O staining. **(D)** Quantitative display of root assay. **(E)** Quantitative display of *en face* assay. Red arrows indicate lesion area. Values are mean ± SD. *N* = 8–9.

Finally, we evaluated atherosclerosis in these mice. We found that, after 3 months on a high fat high cholesterol diet, all mice had lesions in the aortic arch. However, *Lpcat3* KO → *Ldlr* KO mice did not show significant bigger lesions than that of the WT → *Ldlr* KO mice (Figures 7A–E). The male mice also showed same results (Figures 8A–E).

## Discussion

In this study, we have demonstrated that depletion of the *Lpcat3* in macrophages induced a significant 1) reduction of polyunsaturated PCs on cell membrane; 2) induction of M1 macrophages in peritoneal region; and 3) induction of macrophage inflammation through TLR4 and c-Src pathways. However, myeloid cell-specific *Lpcat3* deficiency did not significantly increase atherosclerosis in *Ldlr* KO female and male mice fed a high fat high cholesterol diet for 3 months.

One of the key findings of this study is that LPCAT3 is one of the LPCATs in macrophages (Figure 1C). LPCAT1, LPCAT2, and LPCAT3 can make contribution to PC remodeling in macrophages. LyM-Cre-mediated *Lpcat3* ablation significantly reduced macrophage LPCAT3 activity (80%) (Figure 2B) and reduced polyunsaturated PC levels on the plasma membrane of macrophages (Figure 2C), but not saturated and monounsaturated PCs (Figure 2D).

Another key finding of this study is that LyM-Cre-mediated macrophage *Lpcat3* deficiency has pro-inflammation properties. A previous study indicated that LPCAT3 siRNA significantly increased LPS-mediated inflammatory response in macrophages (14). We found that *Lpcat3* deficiency-mediated macrophage plasma membrane polyunsaturated PC levels reduction can induce TLR4 expression in the lipid rafts (Figure 4A), thereby inducing both MAP kinase and NFκB (Figure 3) activation and promoting inflammatory cytokine productions (Figures 5C,D). Cellular lipids function are important regulators of c-Src activation by altering the recruitment of C-Src to lipid rafts in the plasma membrane (10). Studies have shown a critical role for c-Src in macrophage-mediated inflammatory responses (11). c-Src activates MAP kinases (33–35) and NFκB (36–38). A recent report indicated that c-Src phosphorylation (activation) could participate in macrophage inflammation through NFκB activation and TNFα elevation (32). We found that *Lpcat3* deficiency dramatically increased phosphorylated-c-Src in macrophage lipid rafts (Figure 4C), indicating, besides TLR4 pathway, c-Src pathway might also play an important role in *Lpcat3*-dificiency-mediated effect in macrophages.

It has been reported that acute *Lpcat3* knockdown in hepatocytes and macrophage exacerbated ER stress (14). However, the same group of researchers reported that genetic deletion of *Lpcat3* from the liver did not influence the expression of ER stress markers (39). Previously, we also found that *Lpcat3* deficiency in small intestine had no effect on ER stress markers (40). We found in this study that besides a significant reduction of BIP, IRE1, and PERK had no significant changes (Figure 6C).

We compared our results in this study with a very recent similar study (30). We noticed the following similarity and differences. First of all, we utilized bone marrow derived macrophages from myeloid cell-specific *Lpcat3*-deficient mice, while Thomas et al. utilized fetal liver cells derived macrophages from whole body *Lpcat3* deficient mice. LyM-Cre could only mediate 80% LPCAT3 deficiency (Figures 2A,B) instead of 100% (30). Secondly, both macrophages displayed major reductions in the arachidonate content of phosphatidylcholines (Figure 2C). Thirdly, we found macrophage *Lpcat3* deficiency have no effect on Ac-LDL-mediated cholesterol accumulation as well as cholesterol efflux (Figures 6A,B), while Thomas et al. found that *Lpcat3* deficiency cause an increase in the ratio of free to esterified cholesterol and a reduction in cholesterol efflux in macrophages. Fourthly, we found that macrophage *Lpcat3* deficiency promote inflammation, while the other study did not find changes in macrophage inflammatory response. Finally, we found that myeloid cell-specific *Lpcat3*-deficiency had no significant changes in atherogenesis (Figures 7, 8), while, hematopoietic-specific *Lpcat3*-deficiency promotes atherosclerosis (30).

Although we cannot explain why there was a different outcome of myeloid cell-specific *Lpcat3* deficiency and hematopoietic cell-specific *Lpcat3* deficiency, in terms of mouse atherosclerosis, we speculate that, owning to their hematopoietic origin, *Lpcat3* KO fetal liver also harbored *Lpcat3* KO B-cells, T-cells, mast cells, and granulocytes in the *Lpcat3* KO chimeric mice (*Lpcat3* KO fetal liver cells → *Ldlr* KO) (30). Thus, it is impossible to rule out the possible contributions of these cells in the development of atherogenesis. We prepared myeloid cell-specific *Lpcat3*-deficient mice and transplanted their bone marrow into *Ldlr* KO mice, and then evaluate atherosclerosis in these mice (Figures 7, 8). Nevertheless, we still cannot rule out the contribution of cells besides macrophages in myeloid cell lineage (41).

We also speculate that *Lpcat3* deficiency-mediated changes in macrophage might not be sufficient enough to have an impact on atherogenesity. In PC remodeling system, besides LPCAT3, there are LPCAT1, LPCAT2, and LPCAT4 (13, 15–17). In this study, we indicated that LPCAT1 and LPCAT2 are expressed in macrophages, whereas LPCAT4 expression level is negligible (Figure 1D). Thus, LPCAT1, LPCAT2, and LPCAT3 can all play role in PC remodeling in macrophages. This is different from hepatocytes and enterocyte where LPCAT3 is the major LPCAT (28). LPCAT1 and LPCAT2 are not only involved in PC remodeling activity but also involved in production of platelet activating factor (PAF) (17, 42), a potent proinflammatory phospholipid (43, 44). We found that LPCAT3 deficiency has an impact on downregulation of LPCAT1 and LPCAT2 (Figure 1D) and this could result in reduction of PAF or could be due to regulating lysoPC and/or arachidonic acid (14, 19) availability in macrophages. Further studies are needed to evaluate this LPCAT3 deficiency-mediated effect.

There is limitation of bone marrow transplantation approach of this study. The transplanted macrophage containing LDL receptor. However, LDL receptor contained macrophages have negligible effect on high fat high cholesterol diet induced atherosclerosis in *Ldlr* KO mice (45). Thus, many researchers including us, in the last 20 years, did similar bone marrow transplantation and evaluate atherosclerosis relevance of the genes, which we are interested in, in *Ldlr* KO mice under atherogenic diet.

In conclusion, LPCAT3 contributes to PC remodeling in mouse macrophages and PC composition in macrophage plasma membranes. *Lpcat3* deficiency promotes inflammation. However, such an effect has no significant effect on the development of atherosclerosis.

## Author Contributions

HJ, ZL, and CH did the experiments. X-CJ composed and finalized the manuscript.

### Conflict of Interest Statement

The authors declare that the research was conducted in the absence of any commercial or financial relationships that could be construed as a potential conflict of interest.

## Abbreviations

LPCAT3: lysophosphatidylcholine acyltransferase 3
PC: phosphatidylcholine
KO: knockout
cre: cre recombinase
LPS: lipopolysaccharide
TLR4: toll like 4 receptor
WT: wild type.
